# 

**Published:** 1876-01

**Authors:** 


### Books and Pamphlets Received.

A System of Midwifery, Including the Diseases of Pregnancy and the Puerperal State. By William Leishman, M. D., etc. Second American from the Second Revised English Edition. With Additions by John S. Parry, M. D. Philadelphia: Henry C. Lea, 1875. Buffalo: T. Butler & Son.

The Archives of Dermatology. A Quarterly Journal of Skin and Venereal Diseases. Edited by L. Duncan Bulkley, A. M., M. D. Volume First. New York: G. P. Putnam's Sons.

### Contents of January Number.

### ORIGINAL.

THE BUFFALO

### Medical and Surgical Journal

PUBLISHED MONTHLY,

Containing Original Articles, Reports of Medical Societies and Hospitals, Editorial, Reviews, Correspondence, Army News, etc., etc.; including the usual variety of Medical Periodical Publications. Specimen copies sent on application.

Address Buffalo Medical & Surgical Journal, Buffalo, N. Y.

TERMS OF SUBSCRIPTION, - $3.00 PER YEAR, IN ADVANCE.

